# Sensing Characteristics of SARS-CoV-2 Spike Protein Using Aptamer-Functionalized Si-Based Electrolyte-Gated Field-Effect Transistor (EGT)

**DOI:** 10.3390/bios14030124

**Published:** 2024-02-26

**Authors:** Seonghwan Shin, Sangwon Kim, Wonyeong Choi, Jeonghyeon Do, Jongmin Son, Kihyun Kim, Sungkey Jang, Jeong-Soo Lee

**Affiliations:** 1 Department of Electrical Engineering, Pohang University of Science and Technology (POSTECH), Pohang 37673, Republic of Korea; ssh3290a@postech.ac.kr (S.S.); pathfinder@postech.ac.kr (W.C.); toaru124@postech.ac.kr (J.D.); jmson@postech.ac.kr (J.S.); 2 Department of Life Sciences, Pohang University of Science and Technology (POSTECH), Pohang 37673, Republic of Korea; ksw5758@postech.ac.kr (S.K.); sungkey@postech.ac.kr (S.J.); 3 Division of Electronics Engineering, Jeonbuk National University, Jeonju 54896, Republic of Korea; kihyun.kim@jbnu.ac.kr

**Keywords:** EGT, aptamer, BioFET, SARS-CoV-2, COVID-19

## Abstract

The sensing responses of SARS-CoV-2 spike protein using top-down-fabricated Si-based electrolyte-gated transistors (EGTs) have been investigated. An aptamer was employed as a receptor for the SARS-CoV-2 spike protein. The EGT demonstrated excellent intrinsic characteristics and higher sensitivity in the subthreshold regime compared to the linear regime. The limit of detection (LOD) was achieved as low as 0.94 pg/mL and 20 pg/mL for the current and voltage sensitivity, respectively. To analyze the sensing responses of EGT in detecting the aptamer–SARS-CoV-2 spike protein conjugate, a lumped-capacitive model with the presence of an effective dipole potential and an effective capacitance of the functionalized layer component was employed. The aptamer-functionalized EGT showed high sensitivity even in 10 mM phosphate-buffered saline (PBS) solution. These results suggest that Si-based EGTs are a highly promising method for detecting SARS-CoV-2 spike proteins.

## 1. Introduction

Severe Acute Respiratory Syndrome Coronavirus 2 (SARS-CoV-2) represents a virulent human infectious disease that can cause profound respiratory distress. The World Health Organization (WHO) declared the SARS-CoV-2 outbreak a pandemic and global health emergency on 11 March 2020 [1]. SARS-CoV-2 has a positive-stranded RNA genome that encodes four structural proteins: a spike surface glycoprotein (S), an envelope protein (E), a matrix protein (M), and a nucleocapsid protein (N) [2]. Among these structural proteins, the spike protein is the most reliable biomarker [3,4]. The spike protein is expressed as a trimer on the virus surface, consisting of two subunits, S1 and S2, and plays a crucial role in receptor recognition and cell membrane fusion [5]. The S1 subunit contains a receptor-binding domain (RBD) that recognizes and attaches to the Angiotensin-Converting Enzyme 2 (ACE2) receptor, while the S2 subunit mediates viral cell membrane fusion. The SARS-CoV-2 spike trimer (S1 + S2) protein offers an enhanced biological model compared to the monomeric spike RBD protein or the single S1 subunit.

Real-time polymerase chain reaction (RT-PCR) is recognized as a gold standard for diagnosing SARS-CoV-2 [6,7]. However, this approach is unsuitable for point-of-care testing because it requires specialized laboratories, sophisticated and expensive equipment, and trained personnel. Alternatively, several other methods, including electrochemical techniques [8,9], fluorescence [10,11], and impedance [12,13], have been demonstrated. Among these, biosensor field-effect transistors (BioFETs) have emerged as a promising approach due to their low cost, ultra-sensitivity, and label-free detection [14,15,16]. In conventional BioFETs, the receptor is attached to the channel surface and combined with the target [17,18]. The output drain current of these BioFETs varies with the potential generated by the receptor–target conjugates.

Recently, electrolyte-gated transistors (EGTs) have been fabricated and analyzed using conducting polymer films as their channel materials to detect SARS-CoV-2 [19,20]. The utilization of a significant gate area as a functionalized surface increases the probability of binding events. However, for commercialization, organic EGTs should overcome some disadvantages compared to Si-based EGTs, such as environmental susceptibility, high-voltage operation, and difficulties integrating into existing Si-based devices in complex signal processing systems [21,22].

Aptamers, which are artificially produced single-stranded molecules of DNA or RNA, have high affinity and specificity for binding to target molecules such as proteins or small molecules [23,24]. Due to their smaller size, aptamers are less susceptible to interference from charged molecules in an electrolyte, resulting in a decreased Debye screening effect. It allows aptamers to maintain their binding affinity even in high salt concentrations or complex sample matrices, making them a promising tool for biosensing applications in real-world settings.

Here, we demonstrate the highly sensitive detection of SARS-CoV-2 spike protein (SC2) using aptamer-functionalized Si-EGT. The sensing behaviors under different operating regimes are investigated to achieve higher sensitivity. Furthermore, a lump-capacitive model is applied to analyze the characteristics of the aptamer–SC2 in EGT devices. The influence of buffer concentration and the non-specific binding test are also evaluated.

## 2. Materials and Methods

### 2.1. Fabrication of Si-Based EGT

Here, we demonstrate the ultrasensitive detection of SARS-CoV-2 spike protein (SC2) using aptamer-functionalized EGT. The sensing behaviors under different operating regimes are investigated to achieve higher sensitivity. Furthermore, a lump-capacitive model is applied to analyze the characteristics of the aptamer–SC2 in EGT sensors. The influence of buffer concentration and the non-specific binding test are also evaluated.

**Figure 1:**
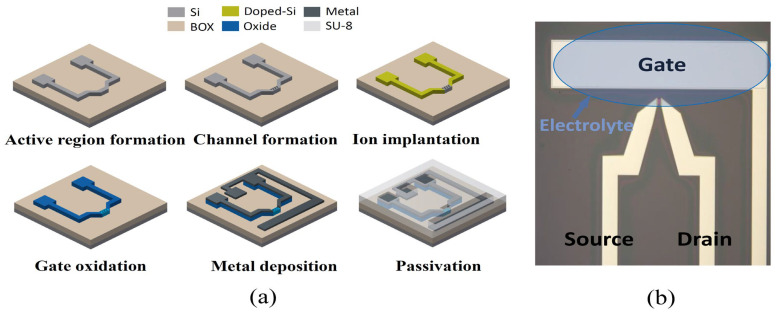
(**a**) Optical microscope image of fabricated Si-based EGT. Inset: SEM image of nanonet channel. (**b**) A schematic of fabrication flow.

Figure 1a shows the fabrication flow of a Si-based EGT using a top-down process. An 8-inch silicon-on-insulator (SOI) wafer (p-type, 10 Ω·cm, (100)), (Soitech, Isere, France) with a 100 nm top-Si and 400 nm buried oxide (BOX) was used. The active region was formed using I-line stepper lithography and an inductively coupled plasma reactive ion etching (ICP-RIE) process (DMS, Taiwan). Then, the channel region was formed using electron beam lithography (E-beam lithography) (Elionix, Tokyo, Japan) and the ICP-RIE process. The ion implantation (As, 5 × 10^15^ cm^−2^) was conducted onto the substrate, followed by rapid thermal annealing (RTA) (Mattson, Fremont, CA, USA) at 1000 °C for 20 s. Then, 5 nm SiO_2_, a gate dielectric, was grown using a high-temperature pyro furnace (Centrotherm, Blaubeuren, Germany) at 800 °C 5 min. Next, the contact pads and transmission lines were formed by depositing Ag/Ti (500 nm/50 nm) using an electron beam evaporator (KVT, Seoul, Republic of Korea) and a conventional lift-off process. Finally, an SU-8 photoresist (Kayaku Advanced Materials Inc., Tokyo, Japan) was passivated on the device to prevent any leakage current flow, excluding the gate (1550 μm × 300 μm) and a channel region (10 μm × 10 μm). Figure 1b shows an optical image of the fabricated device.

### 2.2. Preparation of SARS-CoV-2 Spike Protein and Aptamer

The SARS-CoV-2 spike protein with S1 and S2 subunits was used, which is more similar to the real spike protein than using only the receptor-binding domain (RBD) or S1 subunit. Cells were cultured to produce protein, where Sf9 (Spodoptera frugiperda) cells were cultured in Sf 900 II SFM medium (Gibco, Waltham, MA, USA) with 0.1% penicillin/streptomycin mix and 0.2% amphotericin B (Sigma-Aldrich, St. Louis, MO, USA) in a shaking incubator at 27 °C. A baculovirus expression system was used to produce the spike protein. Baculovirus was generated by transfecting the recombinant baculovirus DNA into SF9 cells. Three days after infection, the medium was centrifuged at 6000 rpm and 10 min conditions to collect supernatant and purified by the Ni-affinity column purification method and size-exclusion chromatography. The aptamer was developed by VIRO-SELEX [25,26]. Several cycles of VIRO-SELEX were performed, and a pool of 11 cycles was chosen to analyze the sequence [27].

### 2.3. Immobilization of Aptamer on EGT

Firstly, devices were rinsed with ethanol and distilled water (DIW), followed by N_2_ blowing. Then, to remove residual contaminants and increase the density of the hydroxyl group, UV/ozone treatment was conducted for 1.5 min. Next, the devices were exposed to 1% 3-aminopropyltriethoxysilane (APTES) (Sigma-Aldrich, St. Louis, MO, USA) vapors for 1 min at 50 °C to cover the surface with an amine group (-NH_2_). After removing the residual APTES by immediate dipping in anhydrous ethanol followed by N_2_ blowing, the devices were immersed in 1 × PBS solution with 2.5% glutaraldehyde (GA) (Sigma-Aldrich, St. Louis, MO, USA) to form an aldehyde group on the surface [28,29,30]. Then, the devices were rinsed with 1 × PBS and DIW followed by N_2_ blowing. Next, to immobilize the aptamer, the devices were exposed to 2.5 μL of 1 × PBS solution containing aptamers for 12 h at room temperature. Finally, the devices were exposed to 0.1% bovine serum albumin (BSA) (Sigma-Aldrich, St. Louis, MO, USA) in 1 × PBS for 90 min at room temperature to block the unreacted active sites to prevent non-specific binding.

### 2.4. Electrical Measurement Set-Up

A semiconductor analyzer (Keithley 4200-SCS, Tektronics, Beaverton, OR, USA) was utilized to characterize the drain current (*I_D_*) and gate voltage (*V_G_*) modulation generated by SARS-CoV-2 responses. All measurements were conducted with a source voltage (*V_S_*) of 0 V, drain voltage (*V_D_*) of 0.1 V, and *V_G_* ranging from 0 V to 1 V with a 0.05 V step. A wide concentration range of SARS-CoV-2 (SC2) from 33 pg/mL to 3.3 μg/mL was prepared in 1 × PBS solution. The measurement sequence was as follows: Initially, the *I_D_*-*V_G_* curve of the aptamer-functionalized devices was characterized in a 0.01 × PBS solution. Next, the device was exposed to an SC2 spike protein solution (1 × PBS, 2.5 μL) for 90 min at room temperature, followed by a rinse. Finally, the *I_D_*-*V_G_* characterization was performed again in 0.01 × PBS solution.

## 3. Results

### 3.1. Electrical Characteristics of Fabricated Si-Based EGT

**Figure 2:**
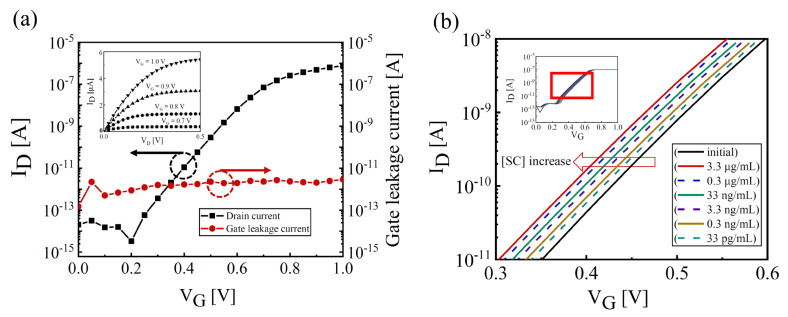
(**a**) A representative intrinsic transfer characteristics (Log (*I_D_*) vs. *V_G_*) and gate leakage characteristic of the fabricated EGT at V_D_ = 0.1 V. The device was characterized in 0.01 × PBS solution. Inset: an output characteristic (*I_D_* vs. *V_D_*) at fixed *V_G_* ranging from 0.7 V~1.0 V. (**b**) A shift in the transfer curve with various [SC2] values. The inset shows the overall transfer curve. A compliance *I_D_* of 100 nA was applied to ensure reproducibility and avoid degradation.

Figure 2a shows a transfer curve (log-scale *I_D_*-*V_G_*) and output curve (*I_D_*-*V_D_*, inset) of the fabricated Si-EGT, demonstrating excellent intrinsic characteristics, including a subthreshold swing (SS ≡ d*V_G_*/dlog (*I_D_*)) of ~80 mV/dec, threshold voltage (*V_TH_*) of 0.65 V, an on–off current ratio of ~10^7^, and a gate leakage current (*I_G_*) of ~10^−12^ A. Figure 2b shows a representative transfer curve of the EGT with various concentrations of [SC2], ranging from the initial amount (no [SC2]) up to 3.3 μg/mL. The increase in [SC2] causes a negative shift in the transfer curves.

### 3.2. Sensing Responses of the EGTs for the SC2 Detection

The current sensitivity (*S_I_*) and voltage sensitivity (*S_V_*) are typically defined as follows [31,32]:

(1) $$S_{I}=\frac{I_{D\_SC2}-I_{D\_AP}}{I_{D\_AP}}$$

(2) $$S_{V}=V_{G_{AP}}-V_{G_{SC2}}$$

where *I_D_AP_* and *I_D_SC2_* are the drain currents at a fixed *V_G_AP_* after aptamer immobilization and after SC2 exposure, respectively, and *V_G_AP_* and *V_G_SC2_* are the gate voltages at a fixed *I_D_AP_* after aptamer immobilization and after SC2 exposure, respectively.

**Figure 3:**
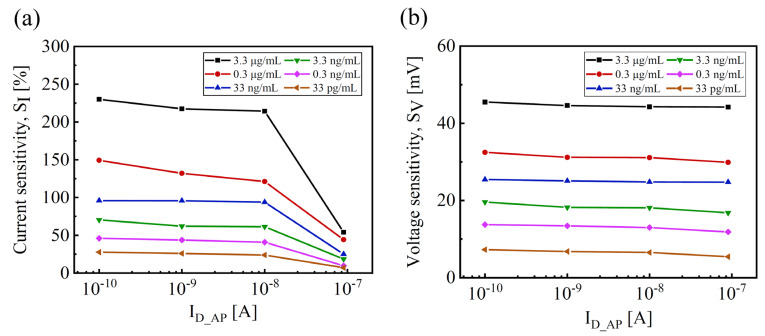
Dependence of sensitivities on the operation regimes: (**a**) average *S_I_* vs. *I_D_AP_* and (**b**) average *S_V_* vs. *I_D_AP_*.

Figure 3 shows the dependence of *S_I_* and *S_V_* on *I_D_AP_*. Both *S_I_* and *S_V_* show different behaviors as the operation shifts from the subthreshold regime (*I_D_* < 10 nA) to the linear regime (*I_D_* > 10 nA) through an increase in *V_G_*. At a given SC2 exposure, *S_I_* shows a constant behavior as *I_D_AP_* decreases in the subthreshold regime, while it radically reduces in the linear regime, as shown in Figure 3a. In contrast, the *S_V_* has a constant behavior independent of *I_D_AP_* values. It suggests that the exposure to SC2 causes little degradation to the devices with a lateral shift in the transfer curve from subthreshold to linear regimes. Thus, the Si-based EGTs should be operated in the subthreshold regime to achieve high sensitivity and low power consumption.

**Figure 4:**
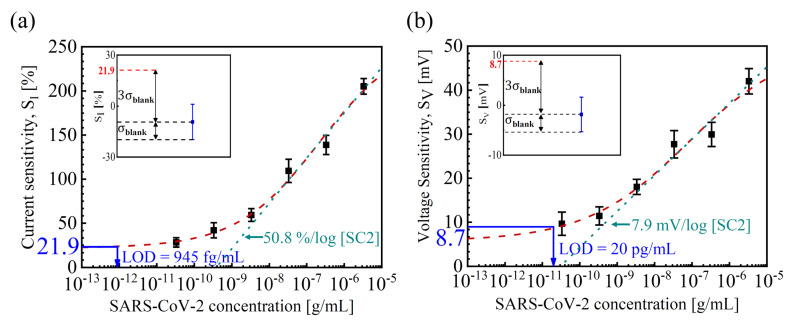
(**a**) *S_I_* and (**b**) *S_V_* vs. [SC2] at *I_D_AP_* = 1 nA. The red dashed lines represent the logistic fitted curves. The inset shows the *S_I_* and *S_V_* values obtained from blank samples without SC2 to calculate the LOD using the three-sigma method.

Figure 4 shows *S_I_* and *S_V_* as a function of [SC2]. The logistic calibration curves were *S_I_* = 376.2 × [SC2]^0.26^/(0.03 + [SC2]^0.26^) and *S_V_* = 72.1 × [SC2]^0.2^/(0.06 + [SC2]^0.2^) [33,34]. The dynamic range was about four orders of magnitude from 33 pg/mL to 3.3 μg/mL of [SC2]. The LOD was determined using the three-sigma method with the fitting sensitivity equation and blank replicate data (1 × PBS without [SC2]) [31,35]. The average of the blank replicate was as low as −9.3% for *S_I_* and −1.84 mV for *S_V_*, respectively, while the standard deviation of the blank replicate was as low as 10.4% for *S_I_* and 3.47 mV for *S_V_*, respectively. The extracted LOD was achieved as low as 945 fg/mL and 20 pg/mL for *S_I_* and *S_V_*, respectively. From the slope of the logistic fitted line in Figure 4, the SC2 sensitivities of 50.8%/log [SC2] for *S_I_* and 7.9 mV/log [SC2] for *S_V_* are extracted, respectively. These values are more than two times higher than the previous results (Table 1).

To further understand the sensing responses of EGT in detecting the aptamer–SC2 conjugate, a lumped-capacitive model with the presence of a dipole layer and a capacitive component was employed [19,36,37]. The binding factor α, defined as the ratio of the aptamer sites bound with the SC2 molecules to the total aptamer sites, can be expressed as [33,36,38]

(3) $$\alpha =\frac{N_{AP\_SC2}}{N_{AP}}=\frac{{\left[SC2\right]}^{n}}{\left(K+{\left[SC2\right]}^{n}\right)}$$

where *N_AP_SC2_* is the density of the aptamer–SC2 conjugates, *N_AP_* is the density of total immobilized aptamers, *K* is related to the dissociation constant of the aptamer–SC2 binding, and *n* is a slope factor that indicates the cooperativity of SC2 binding.

After introducing SC2 to the aptamer-functionalized surface, it generates an effective dipole moment (*V_DP_EFF_*) and an effective capacitance (*C_FN_EFF_*). *V_DP_EFF_* can be expressed as follows [20,36,37]:

(4) $$V_{DP\_EFF}=\frac{\alpha \cdot N_{AP}\cdot P_{DP}}{\varepsilon_{DP}}≅V_{DP\_GT}$$

where *P_DP_* is the perpendicular component of the dipole moment, *ε_DP_* is the permittivity of the dipole layer, and *V_DP_GT_* is the dipole potentials at the gate electrode. *V_DP_EFF_* is mainly determined from the gate electrode since the gate area is ~5000 times larger than the channel area in the fabricated EGT device. The established *V_DP_EFF_* changes the flatband voltage, causing a shift in the *I_D_* vs. *V_G_* curve. The positive *V_DP_EFF_* reduces *V_TH_* and shifts the transfer curve toward the negative *V_G_* direction.

*C_FN_EFF_* is related to the biomaterials on the channel and can be expressed as follows [36]:

(5) $$C_{FN\_EFF}=C_{SC2\_CH}+(C_{SC2\_CH}+C_{AP\_CH})\frac{K}{{\left[SC2\right]}^{n}}$$

where *C_SC2_CH_* and *C_AP_CH_* are the SC2 and aptamer capacitances per unit area at the channel.

Then, *S_V_* is calculated using *V_DP_EFF_* and *C_FN_EFF_* as follows:

(6) $$S_{V}=V_{DP\_EFF}+\frac{Q_{S}}{C_{FN\_EFF}}$$

where *Q_S_* is the total charge density in the channel.

**Figure 5:**
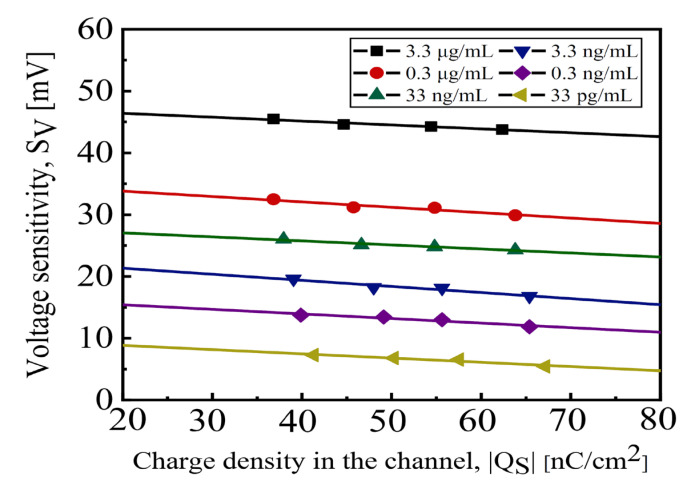
|*Qs*| vs. *S_V_* with various values of [SC2].

Figure 5 shows the |*Q_S_*| versus *S_V_* characteristics for different [SC2] values. For an n-type channel, the negative *Q_S_* can be determined by summing the depletion charge, inversion charge, and interface trap charge in the channel [39,40]. *V_DP_EFF_* and *C_FN_EFF_* are obtained from the y-intercept and reciprocal slope of the curves, respectively.

**Figure 6:**
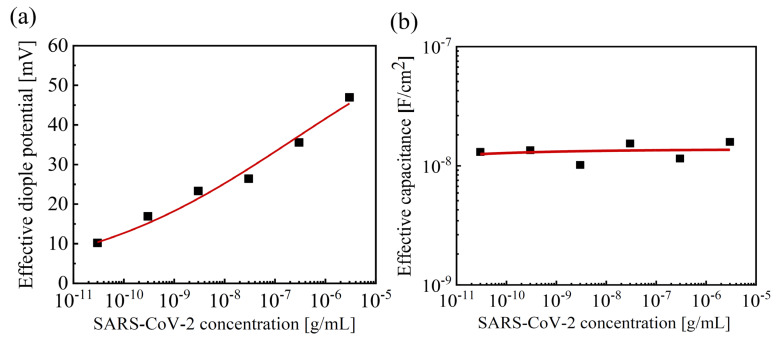
(**a**) The extracted effective dipole potential *V_DP_EFF_* and (**b**) effective capacitance *C_FN_EFF_* with various [SC2].

Figure 6a shows the relationship between *V_DP_EFF_* and [SC2]. The positive *V_DP_EFF_* is clearly proportional to [SC2], and the logistics calibration curve of *V_DP_EFF_* is obtained as *V_DP_EFF_* = 72.7 × [SC2]^0.2^/(0.047 + [SC2]^0.2^). By utilizing equations (3), the binding factor *α* can be calculated as *α* = [SC2]^0.2^/(4.7 × 10^−2^ + [SC2]^0.2^) with *K* = 4.7 × 10^−2^ and *n* = 0.2. A lower *K* value of the aptamer reaction, compared to the previous protein detection (*K* = 1.84 × 10^−3^), indicates that the aptamer conjugate is more prone to dissociation than the peanut protein conjugates [36]. In Figure 6b, the extracted *C_FN_EFF_* is fitted as *C_FN_EFF_* = 1.37 × 10^−8^ − 9.4 × 10^−12^ × [SC2]^−0.2^ ≈ 1.37 × 10^−8^ using the extracted *K* and *n* values. The [SC2] capacitance generates a constant *C_FN_EFF_* for various [SC2]. Thus, compared to previous protein detection [36], the binding characteristics in the channel are negligibly involved in the overall aptamer detection.

Debye screening is another crucial factor that can affect the sensing performance of BioFETs. It refers to the phenomenon in which charged ions in an electrolyte shield the electric charge of the conjugates [41,42,43]. The Debye length is the characteristic length scale over which the electrical potential of a charged molecule decays exponentially due to the surrounding ions in an electrolyte. When the Debye length is comparable to the distance between the channel surface and the conjugates, the electrostatic interaction is significantly weakened due to the shielding effect of the charged ions in the solution [44].

**Figure 7:**
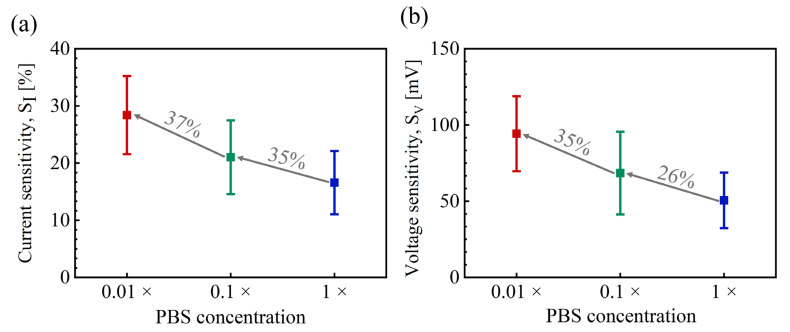
Dependence of (**a**) *S_I_* and (**b**) *S_V_* on the PBS concentrations. *S_I_* was extracted at *I_D_AP_* = 1 nA and [SC2] = 33 ng/mL.

To assess the impact of Debye screening on the responses to SC2, three different PBS solutions (0.01 ×, 0.1 ×, and 1 × PBS) were prepared by diluting 1 × PBS with DI water. Figure 7 shows the measured *S_I_* and *S_V_* at different PBS concentrations using an exposure of 33 ng/mL SC2. Both sensitivities increased as the PBS concentration decreased. The *S_I_* and *S_V_* values were approximately 80% higher in 0.01 × PBS buffer than in 1 × PBS solution. Interestingly, our devices were capable of detecting [SC2] even in 1 × PBS solution, despite the larger [SC2] used compared to other SARS-CoV-2 spike proteins that typically consist of only a smaller S1 subunit and RBD [14,45].

**Figure 8:**
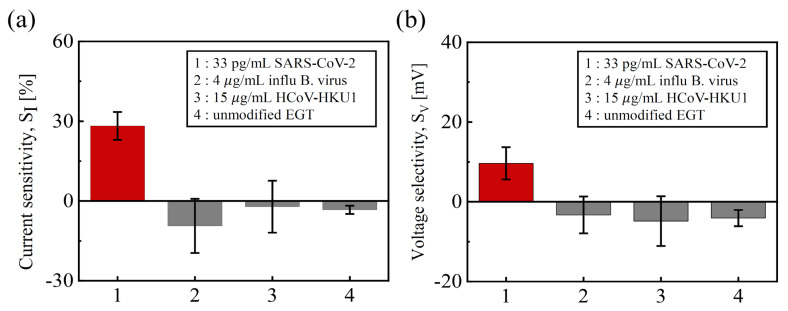
Selectivity tests of (**a**) *S_I_* and (**b**) *S_V_* with 4 μg/mL influenza B virus, 15 μg/mL HCoV-HKU1, and unmodified EGTs.

Figure 8 presents non-specific control experiments to confirm the selectivity to SC2. Various test samples, such as influenza B virus HA (hemagglutinin) protein and human coronavirus (HCoV-HKU1) spike protein, were exposed to high enough concentrations to confirm the specificity of the SARS-Cov-2 spike protein. The *S_I_* and *S_V_* values obtained from these biomolecules were sufficiently low and below 33 pg/mL of SC2, indicating that the sensitivity is specific to SC2 binding. In addition, when exposed to 3.3 μg/mL of SC2, the unmodified EGT exhibited negligible *S_I_* and *S_V_*, indicating that the SC2 aptamers were successfully immobilized and could be used to evaluate the surface functionalization process.

Table 1 compares the sensing performance of the EGT with previously reported sensors for detecting SC2. The Si-based EGT shows a significant improvement in both dynamic range and sensitivity.

**Table 1:** Comparison between various SC2 sensors.

Sensor Type	Biomarker	Dynamic Range	Sensitivity from *S_I_* (%/log [SC2])	Limit of Detection	Ref.
Paper-based electrochemical sensor	RBD	10^3^ (1 ng/mL to 1 μg/mL)	10.7	1 ng/mL	[46]
CNT-FET sensor	S1	5 × 10^4^ (0.1 fg/m to 5 pg/mL)	3.8	4.12 fg/mL	[47]
Graphene-FET sensor	S1	10^4^ (1 fg/mL to 10 pg/mL	16	1 fg/mL	[14]
Electrical-double-layer (EDL)-gated FET sensor	N protein	10^3^ (0.4 ng/mL to 400 ng/mL	4.6	0.34 ng/mL	[48]
Organic electrochemical transistor immuno-sensor	RBD	10^6^ (1.4 pg/mL to 1.4 μg/mL)	1.6	1.4 pg/mL	[49]
Si-based EGT	S1 + S2	10^5^ (33 pg/mL to 3.3 μg/mL)	50.8	945 fg/mL	This work

## 4. Conclusions

We have successfully demonstrated the highly sensitive detection of SARS-CoV-2 spike protein using aptamer-functionalized Si-based EGTs. A lumped-capacitive model was utilized to analyze the aptamer–SC2 conjugates on the sensing performances in terms of dipole moments and capacitive components. The fabricated EGT showed higher sensitivities in the subthreshold regime, achieving LODs of 0.94 pg/mL and 20 pg/mL for current and voltage sensitivity, respectively. In addition, the EGT showed high sensitivity even in 1 × PBS solution. These results suggest that the Si-based EGTs using advanced microfabrication technology are promising in detecting SARS-CoV-2 spike proteins.

## Data Availability

Data are contained within the article.
